# A Peptide Inhibitor of the Human Cytomegalovirus Core Nuclear Egress Complex

**DOI:** 10.3390/ph15091040

**Published:** 2022-08-23

**Authors:** Sewar Alkhashrom, Jintawee Kicuntod, Katharina Stillger, Tamara Lützenburg, Christian Anzenhofer, Ines Neundorf, Manfred Marschall, Jutta Eichler

**Affiliations:** 1 Department of Chemistry and Pharmacy, Friedrich-Alexander-Universität Erlangen-Nürnberg, 91058 Erlangen, Germany; 2 Institute of Clinical and Molecular Virology, Universitätsklinikum Erlangen, Friedrich-Alexander-Universität Erlangen-Nürnberg, 91054 Erlangen, Germany; 3 Department of Chemistry, Institute of Biochemistry, University of Cologne, 50674 Köln, Germany

**Keywords:** human cytomegalovirus, nuclear egress complex (NEC), antiviral peptide, cellular uptake, nuclear uptake, cell penetrating peptide (CPP), nuclear localization signal (NLS)

## Abstract

The replication of human cytomegalovirus (HCMV) involves a process termed nuclear egress, which enables translocation of newly formed viral capsids from the nucleus into the cytoplasm. The HCMV core nuclear egress complex (core NEC), a heterodimer of viral proteins pUL50 and pUL53, is therefore considered a promising target for new antiviral drugs. We have recently shown that a 29-mer peptide presenting an N-terminal alpha-helical hook-like segment of pUL53, through which pUL53 interacts with pUL50, binds to pUL50 with high affinity, and inhibits the pUL50–pUL53 interaction in vitro. Here, we show that this peptide is also able to interfere with HCMV infection of cells, as well as with core NEC formation in HCMV-infected cells. As the target of the peptide, i.e., the pUL50–pUL53 interaction, is localized at the inner nuclear membrane of the cell, the peptide had to be equipped with translocation moieties that facilitate peptide uptake into the cell and the nucleus, respectively. For the resulting fusion peptide (NLS-CPP-Hook), specific cellular and nuclear uptake into HFF cells, as well as inhibition of infection with HCMV, could be demonstrated, further substantiating the HCMV core NEC as a potential antiviral target.

## 1. Introduction

The human cytomegalovirus (HCMV) is a worldwide distributed human pathogen, which belongs to the beta-subfamily of the herpesviruses. Infection of immunocompetent individuals is mostly asymptomatic, accompanied by productive replication of the virus, before establishing a lifelong viral latency. In immunosuppressed individuals, such as solid organ transplant recipients, as well as AIDS and cancer patients; however, HCMV infection can result in severe systemic or even life-threatening disease [1,2,3]. Most importantly, HCMV is the most frequent congenital virus infection, which often results in severe conditions, including microcephaly, as well as damage to the developing central nervous system of the newborn, leading to hearing loss and mental retardation [4,5,6]. Established anticytomegaloviral drugs include ganciclovir, its valine ester derivative valganciclovir, foscarnet and cidofovir, which all target the viral DNA polymerase [3,7]. An alternative mechanism has been reported for the prophylactic anti-HCMV drug letermovir/Prevymis^®^, whose antiviral activity is based on inhibition of a component in the terminase complex [8,9]. Furthermore, the first inhibitor of the viral protein kinase pUL97, termed maribavir, has recently been approved for the therapy of drug-refractory conditions of HCMV infection [10,11]. Most of these drugs, however, are associated with side effects, limited bioavailability, as well as the emergence of drug-resistant virus variants [12,13]. Therefore, biomedical research is focused on novel antiviral strategies against HCMV infection, which target alternative components of the virus replication cycle. 

Replication of herpesviruses involves a process termed nuclear egress [14,15,16], which involves a range of protein interaction and phosphorylation events, resulting in the formation of the nuclear egress complex (NEC), which, through distortion of the nuclear lamina, enables transport of newly formed viral capsids from the nucleus into the cytoplasm [17,18,19,20,21]. In HCMV replication, NEC formation is initiated by the interaction of two viral proteins, pUL53 and pUL50 [22], which assemble into the core NEC as a scaffold for further protein association and NEC-specific activities [17]. 

As the nuclear egress of herpesviruses represents an essential and rate-limiting step in virus replication [23], it is highly relevant in the context of novel antiviral strategies. In particular the core NEC of HCMV has been proposed as a target for HCMV inhibitors [23]. We have recently identified, through the screening of the Prestwick Chemical Library^®^, an inhibitory small molecule of the HCMV core NEC [24]. This molecule was shown to inhibit HCMV infection of cells, and to disrupt the pUL50–pUL53 nuclear rim co-localization [24], supporting the concept of the core NEC as a promising novel drug target for the therapy of HCMV, and possibly other herpesviral infections as well.

**Figure 1:**
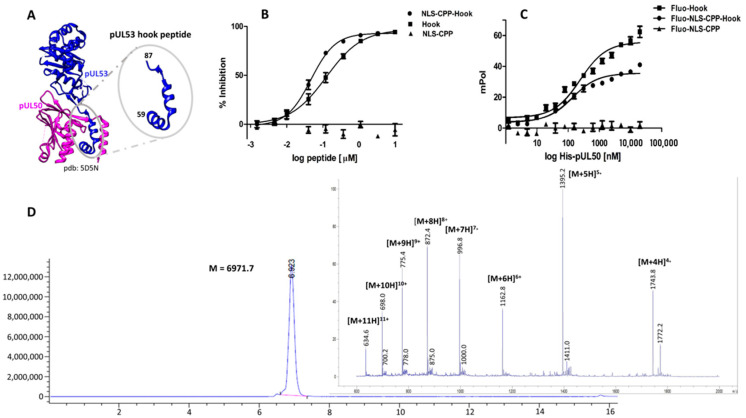
(**A**), Crystal structure of pUL53 (blue) in complex with pUL50 (purple), pdb code 5D5N. (**B**), Inhibition of the HCMV pUL50–pUL53 interaction by the hook peptide, the NLS-CPP-hook fusion peptide, and NLS-CPP. (**C**). Binding of the three peptides to pUL50. (**D**), HPLC chromatogram and mass spectrum of the fusion peptide NLS-CPP-hook. See Materials and Methods for experimental details.

In a complementary, structure-based approach, we have designed and characterized a 29-mer peptide that presents the binding site of HCMV pUL53 for its interaction with pUL50, which is located in an alpha-helical hook-like region at the N-terminus of pUL53, and which provides approximately 80% of the interaction interface with pUL50 (Figure 1A) [25,26]. This peptide was shown to inhibit the interaction of recombinant, soluble core NEC proteins pUL50 and pUL53, at nanomolar concentrations [23,26]. Furthermore, alanine and D-amino acids scans of the pUL53 hook peptide were used to explore the determinants of its interaction with pUL50 [23]. We now asked the question whether the peptide is able to interfere with core NEC formation also in the context of HCMV infection, which would translate into an antiviral effect. As the core NEC is localized at the inner nuclear membrane of the infected cell, an inhibitor of this interaction has to be able to enter the cell, as well as to translocate into the nucleus. Unlike small molecules, which can readily penetrate membranes [27], larger molecules, such as the pUL53 hook peptide, typically need to be equipped with translocation domains that facilitate their cellular and nuclear uptake. A well-established mode of transporting molecular cargo into cells is by means of small cationic or amphipathic cell penetrating peptides (CPPs), which are capable of crossing cell membranes [28,29,30,31,32,33,34]. Translocation of peptides and proteins from the cytoplasm into the nucleus, on the other hand, can be facilitated by nuclear localization signal (NLS) peptides [35,36]. Therefore, we set out to fuse the pUL53 hook peptide to a CPP, as well as to an NLS peptide, and to use this fusion peptide to explore the antiviral potential of the pUL53 hook peptide.

## 2. Results and Discussion

### 2.1. Design and Evaluation of the pUL53 Hook Fusion Peptide

In order to enable the pUL53 hook peptide to enter the cell nucleus, where its target, i.e., the core NEC, is localized, the peptide had to be equipped with appropriate translocation domains. An established CPP presenting residues 47–59 of the human immunodeficiency virus type 1 (HIV-1) Tat protein [37,38,39,40] was used for cellular uptake, in conjunction with the pUL53 analogous nuclear localization signal peptide (NLS) presenting residues 18–27 of pUL53, which facilitates nuclear import of this nucleoplasmic protein, and which has been shown to also translocate other proteins into the nucleus [41]. 

The resulting fusion peptide, termed NLS-CPP-Hook, was generated through solid-phase peptide synthesis (Figure 1D, see Table 1 for peptide sequences). The three segments of this 54-mer peptide were separated from each other by 8-amino-3,6-dioxaoctanoic acid (Aoa) residues. In order to address the possible impact of the CPP and NLS sequences on the inhibitory activity of the hook peptide, a peptide presenting only these parts (NLS-CPP) was used as a negative control, while a peptide presenting only the pUL53 hook sequence (Hook peptide) served as reference. All three peptides were evaluated regarding their ability to inhibit the pUL50-pUL53 interaction, as well as their affinity to pUL50, using previously reported inhibition [23,24,26] and binding [23] assays (Figure 1B,C, Table 1). As shown in Figure 1B, the inhibitory effect of the NLS-CPP-Hook (IC_50_ = 47 nM) on the pUL50-pUL53 interaction is very similar to that of the Hook peptide alone (IC_50_ = 125 nM), indicating that fusion to the NLS and CPP sequences does not dramatically affect the activity of the Hook peptide. The NLS-CPP peptide lacking the pUL53 hook peptide, on the other hand, was completely inactive, indicating that it does not interact with neither pUL50, nor with pUL53, and re-confirming the previously established specificity of the inhibitory effect of the hook peptide [23]. These results were confirmed in a fluorescence polarization binding assay using fluoresceinylated peptides, in conjunction with recombinant pUL50 (Figure 1C, Table 1), where again the affinity to pUL50 of the hook peptide (Fluo-Hook, Kd = 228.4 nM) and the Fluo-NLS-CPP-Hook fusion peptide (Kd = 122.4 nM) were very similar, while the Fluo-NLS-CPP peptide alone did not bind to pUL50 at all, illustrating the selectivity of the interaction of the pUL53 hook peptide with pUL50. As fusion to the CPP and NLS sequences was shown not to interfere with the binding behavior and inhibitory activity of the pUL53 hook peptide, we felt confident to proceed with the NLS-CPP-hook peptide in cellular and nuclear uptake and virus neutralization experiments, respectively. 

### 2.2. Cellular and Nuclear Peptide Uptake

**Figure 2:**
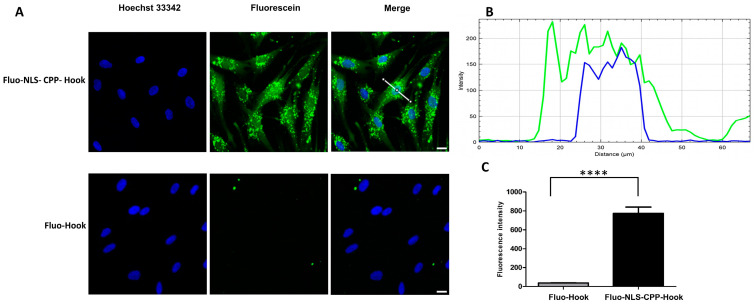
Cellular and nuclear uptake of the pUL53 hook peptide. (**A**), Live cell fluorescence microscopy of HFF cells treated with 5 µM fluoresceinylated hook peptide (Fluo-Hook) and its NLS-CPP fusion peptide (Fluo-NLS-CPP-Hook) for two hours. Green: Fluorescein-labeled peptide, Blue: nuclear Hoechst 33342 dye, scale bar: 20 µm. (**B**), Corresponding line intensity profile of the nucleus (blue) and Fluo-NLS-CPP-Hook (green). (**C**), Flow cytometry analysis of HFF cells treated with 1 µM Fluo-hook and Fluo-NLS-CPP-hook, respectively, for 30 min. See Materials and Methods for experimental details. Statistical significance was determined using the student´s *t*-test (**** *p* < 0.0001).

Peptides used for cellular and nuclear uptake experiments were fluoresceinylated at the N-terminus, enabling visualization through live cell fluorescence microscopy, as well as quantification by flow cytometry. The fluoresceinylated peptides were tested in conjunction with HFF cells, which were also used for the subsequent confocal imaging, cytotoxicity and virus neutralization assays. As some alpha-helical peptides, such as the hook peptide, have been shown to be able to penetrate membranes on their own [42,43], we examined translocation of the fluoresceinlyated hook peptide (Fluo-hook) lacking the CPP and NLS moieties, as well as the fluoresceinylated NLS-CPP-hook peptide (Fluo-NLS-CPP-hook). While Fluo-NLS-CPP-hook was efficiently taken up into the cells, as well into the nucleus (Figure 2A,B), internalization of Fluo-hook was significantly weaker, illustrating the strong effect of the translocation moieties in facilitating cellular and nuclear uptake of the hook peptide. This observation is in perfect agreement to recent studies, in which we have explored and proven the benefit of combining CPP and NLS for efficient drug delivery into the nucleus [44]. 

### 2.3. Delocalization of pUL53 from the Nuclear Rim

**Figure 3:**
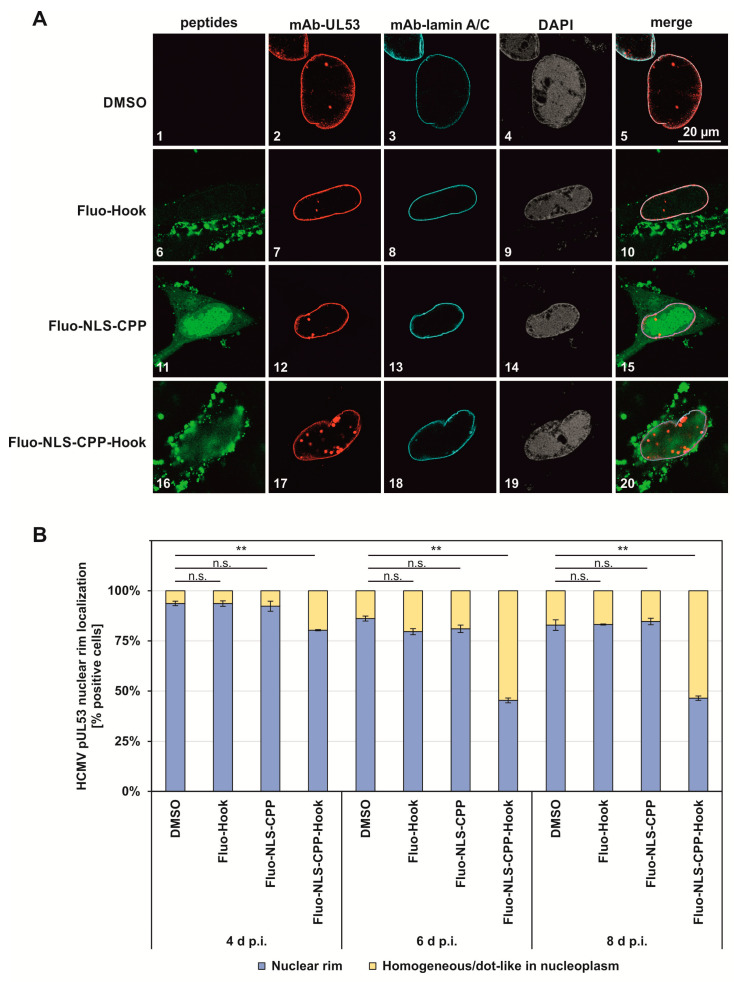
Effect of peptides on pUL53 nuclear rim localization. (**A**). Confocal imaging (indirect immunofluorescence staining) of viral pUL53 and cellular lamin A/C. Counterstainings of the fluoresceinylated peptides and the nuclei (DAPI) are shown and a merge of all signals is given. (**B**). Microscopic quantitation was performed by visual inspection and counting of cells showing a peptide-mediated reduction in pUL53 nuclear rim localization. See Materials and Methods for experimental detail. Statistical significance was determined using the student’s *t*-test (** *p* 0.01; n.s., *p* > 0.05).

The viral protein pUL50 is anchored to the inner nuclear membrane of HCMV infected cells, where it heterodimerizes with soluble pUL53, forming a nuclear rim. Therefore, an inhibitory effect of the hook peptide on the pUL50–pUL53 interaction in infected cells would be expected to result in a delocalization of pUL53 from the inner nuclear membrane and a distortion of the nuclear rim. This effect of the pUL53 hook peptide was investigated by indirect immunofluorescence staining and confocal imaging (Figure 3A, panels 2, 7, 12, 17). The rim signal pattern that illustrated the ordinary structure of nuclear rim formation was monitored via control staining with a nuclear lamin A/C-specific antibody (Figure 3A, panels 3, 8, 13, 18). Fluoresceinylated peptides are visualized in panels 6, 11 and 16. It should be noted that Fluo-NLS-CPP-Hook (panel 17 and 20), but not the hook peptide lacking the NLS-CPP moiety (Fluo-Hook, panel 7 and 10), nor the NLS-CPP moiety without the hook peptide (Fluo-NLS-CPP, panel 12 and 15) were able to alter the fine-localization of pUL53. This effect is demonstrated by the disruption of the nuclear rim signal, as evidenced by the appearance of dot-like speckles in the nucleoplasm. The structure of the cell nuclei (counterstained by DAPI, panels 4, 9, 14, 19) did not show relevant signs of alterations by the three peptides. 

In order to quantitate this inhibitory effect, three areas of positive cells (192 cells on average, counted as technical triplicates), as contained on two biological replicates of slides, were used for evaluation. As a result, this microscopic inspection of cells clearly indicated a specific rim-disturbing effect of Fluo-NLS-CPP-Hook (Figure 3B), demonstrating a statistically significant reduction in the typical pUL53 nuclear rim localization towards a nucleoplasmic localization pattern (dot-like or homogeneous). This inhibitory effect increased over time of infection to 80%, 45% and 46%, respectively. The other two peptides only showed marginal effects that were not statistically significant (n.s.). Thus, these data indicate an inhibitory effect of the peptide Fluo-NLS-CPP-Hook on the NEC formation in HCMV-infected cells.

### 2.4. Antiviral Activity and Cytotoxicity

**Figure 4:**
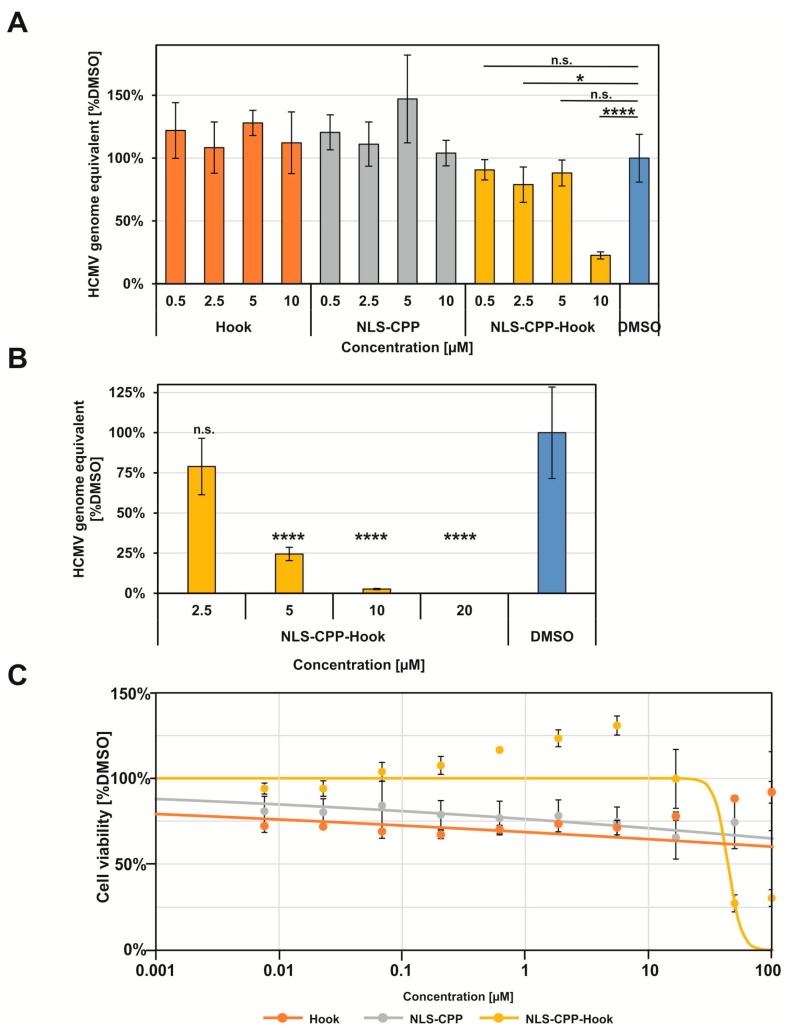
Anti-HCMV activity in the genome-specific qPCR assay (**A**,**B**) and lack of cytotoxicity (**C**) of peptides. Error bars present standard deviations calculated from three or four different experiments. Statistical significance was determined using the Student’s *t*-test (**** *p* 0.0001; * *p* 0.05; n.s., *p* > 0.05). See Materials and Methods for experimental detail.

Having established the core NEC inhibitory effect of the pUL53 hook peptide fused to the CPP and NLS moieties, as demonstrated by its impact on pUL53 nuclear rim localization, we then addressed its inhibitory effect on HCMV replication efficiency. For this purpose, HFF cells were infected with HCMV AD169, and treated with the respective peptides. Efficiency of virus production was assessed by measuring viral genome equivalent through HCMV-specific qPCR, in the supernatants of infected cells, at time points 4, 6 and 8 d p.i. (Figure S1). Using this method, an inhibitory effect on virus replication could be detected only for NLS-CPP-Hook (Figure 4A). Unlike the NLS-CPP-Hook peptide, the two control peptides NLS-CPP and Hook had no significant effect on HCMV infection at concentrations up to 10 µM (Figure 4A), illustrating the selectivity of the inhibitory activity of the hook peptide (NLS-CPP is inactive), as well as the fact that translocation moieties are required to enable cellular and nuclear uptake of the hook peptide, as the hook peptide by itself is unable to interfere with HCMV replication. Furthermore, the antiviral effect of the NLS-CPP-Hook peptide was shown to be concentration dependent (Figure 4B), with an EC_50_ value of 5.6 µM (Table 2). As this value is approximately 45-fold higher than the IC_50_ value of the hook peptide for the inhibition of the pUL50–pUL53 interaction in vitro (Table 1), it appears likely that the translocation of the peptide into the cell, as well as into the nucleus, is not yet optimal. Additionally, the peptide may not be completely stable in the medium used for the infection assay. While these considerations indicate directions for optimization of the fusion peptide, the data shown here clearly demonstrate that the inhibitory effect of the hook peptide on the interaction of recombinant soluble pUL50 and pUL53, translates into an antiviral effect. This result further substantiates the postulation of the core NEC of HCMV, and possibly other herpesviruses as well, as a potential antiviral target. 

Furthermore, addressing the question whether the observed antiviral effect of the NLS-CPP-Hook peptide is possibly a result of a general cytotoxicity of the peptide, rather than a specific interference with virus replication, we assessed the impact of the peptide on cell viability outside the context of an HCMV infection, using standard Neutral Red assay, which was performed on day 7 of peptide treatment (Figure 4C). While the NLS-CPP-Hook peptide was shown to be toxic to HFF cells at higher concentrations, the CC_50_ value (44.2 µM) for cytotoxicity is approximately eight-fold higher than the EC_50_ value (5.6 µM) for the antiviral effect (Table 2). Consequently, the antiviral effect of NLS-CPP-Hook at concentrations below 20 µM can be considered specific, i.e., not a result of a general cytotoxicity of the peptide. 

## 3. Materials and Methods

### 3.1. Peptide Synthesis

Peptides were synthesized as C-terminal amides by Fmoc/tBu-based solid-phase synthesis, on TentaGel S RAM resin (100 mg, 0.23 mmol/g) using an automated multiple peptide synthesizer (ResPep from Intavis Inc.), as previously described [45]. After the final amino acid coupling, the N-terminus was either acetylated (Hook peptide, NLS-CPP and NLS-CPP-Hook), or fluoresceinylated (Fluo-Hook, Fluo-NLS-CPP and Fluo-NLS-CPP-Hook) by coupling to 5(6)-carboxyfluorescein. Peptides were cleaved from the resin by use of trifluoroacetic acid (TFA)/water/phenol/thioanisole/triisopropylsilane 82.5:5:5:5:2.5, precipitated in cold tert-butyl methyl ether, extracted with water, and lyophilized. Peptides were purified by preparative HPLC (Phenomenex Kinetex C18 column, 100 × 21.2 mm, flow rate 20 mL/min, gradient of acetonitrile in H_2_O (both containing 0.1% TFA, 30–45% over 8 min). Stock solutions of purified peptides were prepared at 10 mM in DMSO. For analytical data of purified peptides (LC/MS) see Supplementary Materials (Figure S2).

### 3.2. pUL50 Binding and pUL50-pUL53 Inhibition Assay

*Binding assay*. N-terminally fluoresceinylated peptides were diluted in the assay buffer (0.1 M PB, 150 mM NaCl, 1 mM TCEP, pH 7.2, 0.01% Tween 20) to provide a 25 nM solution. Peptide solutions were incubated with recombinant pUL50 [26] at two-fold serial dilutions, starting at 20 µM. Fluorescence polarization was measured at 485 nm (excitation) and 535 nm (emission).

*Inhibition assay*. Recombinant pUL50 and pUL53 were produced and purified as previously described [26]. High binding Immulon microtiter plates were coated with 100 µL pUL53 (1 µg/mL) in sodium carbonate buffer pH 9.5, overnight at 4 °C. After blocking with 200 µL 1% BSA in 0.1 M phosphate buffer, pH 7.2, for two hours, plates were incubated with 50 µL peptide in serial dilutions, starting at 10 µM, together with 50 µL His-tagged pUL50 (0.78 µg/mL) for two and a half hours. His-tagged pUL50 was detected using anti-His-HRP (Merck, Darmstadt, Germany, 1:100,000). All proteins and antibodies were in 0.1 M phosphate buffer, pH 7.2, containing 0.1% BSA and 0.01% Tween 20. Plates were washed four times with 0.01% Tween 20 in 0.1 M phosphate buffer, pH 7.2, after each incubation step. Plates were developed with o-phenylenediamine (OPD) (1 mg/mL) in the presence of 0.03% H_2_O_2_ for approximately 5 min in the dark. After the reaction was stopped with 50 µL 2 M H_2_SO_4_, absorbance was read at 492 nm. IC_50_ values were determined from the % inhibition data using the program GraphPad. Inhibition was calculated according to the following formula:

% Inhibition = [1 − (As_ample_ − A_blank1_)/(A_100%_ − A_blank2_)] × 100

in which “100%” is a sample without inhibitor, “blank1” is a sample without pUL53, and “blank2” is a sample without pUL53 and without inhibitor.

### 3.3. Cellular and Nuclear Uptake of Peptides

For flow cytometry analysis, human foreskin fibroblasts (HFF, ATCC: SCRC-1041™) were seeded in 24-well plates. Upon reaching 80–90% confluency, cells were treated with 1 µM Fluo-labeled peptide solutions diluted in serum-free medium for 30 min at 37 °C. Afterwards, cells were washed with PBS and detached from the plate using phenol red-free trypsin and resuspended in phenol red-free medium. The cell suspension was analyzed using the guava easyCyte HT flow cytometer using the GRN-B (525/530) channel.

For live imaging microscopic analysis, HFF were seeded into 8-well plates (Ibidi). On the next day, cells were treated with 5 µM Fluo-labeled peptide diluted in serum-free medium for 2 h at 37 °C. The nuclei were stained with Hoechst 33342 for the last 10 min of the total incubation time. After removal of the medium, trypan blue was used to quench external fluorescence. Cells were washed three times with serum-free medium, and complete medium was added for microscopy analysis. Microscopy was performed using a Keyence BZ-X800E microscope equipped with a 20X objective. Images were processed using Fiji software. 

### 3.4. Cell Culture and Virus Infection

Primary human foreskin fibroblasts (HFFs) were cultivated in minimal essential medium (MEM). Cell culture media were supplemented with 1× GlutaMAXTM (35050038, Thermo Fisher Scientific), 10 g/mL gentamicin and 10% fetal bovine serum (FBS, F7524, Sigma Aldrich). All cells were incubated at 37 °C, 5% CO2 and 80% humidity. For infection with human cytomegalovirus (HCMV strain AD169), 2 × 10^5^ HFFs were seeded in 12-well plates for 1 day (d) prior to infection at a multiplicity of infection (MOI) of 0.1. After 90 min of viral absorption, cells were incubated with peptides in the culture media media, which was supplemented with 2.5% FBS, 1× GlutaMAX^TM^ and 10 g/mL gentamicin, at the indicated concentrations at 37 °C for 4–8 days. Viral supernatants were collected at 4, 6 and 8 days post-infection (d.p.i.).

### 3.5. Indirect Immunofluorescence Assay and Confocal Laser-Scanning Microscopy

HFFs were cultivated in 6-well plates on cover slips, and used for HCMV infection at MOI of 0.1. After 90 min of viral absorption cells were treated with 6 µM of the indicated fluoresceinylated peptides, and incubated at 37 °C. Peptides contained in the culture media were refreshed at 2, 5 and 7 d p.i., and cells were fixed at 8 d p.i. with 10% formalin (8 min, room temperature). Afterwards, HFFs were permeabilized using 0.2% Triton X-100 in PBS and blocked with cohn II. Cells were incubated with viral pUL53 and cellular lamin A/C primary antibodies for 60 min at 37 °C prior to double stain with secondary antibodies conjugated Alexa Fluor^®^ 555 and Alexa Fluor^®^ 647. The nucleus was counterstained with DAPI Vectashield mounting medium. Data for immunofluorescence were collected using a TCS SP5 confocal laser-scanning microscope (Leica Microsystems, Wetzlar, Germany). Images of a confocal plane were taken with a line average of 3 at magnification of 1024 × 1024.

### 3.6. Quantitative Polymerase Chain Reaction (qPCR)

HFFs were seeded in 12-well plates, used for HCMV infection at a MOI of 0.1 and treated with the indicated concentrations of each peptide. Peptides contained in the culture media were refreshed at 2, 5 and 7 d p.i., and viral supernatants were collected at 8 d p.i. for an assessment of the viral genomic load by qPCR. The supernatants were centrifuged at 1500× *g* and digested with proteinase K at 56 °C for 1 h to release viral particles. The reactions were stopped at 95 °C for 5 min. The amount of extracellular viral genomic loads was assessed in 5 mL of each sample by real-time PCR (TaqMan-PCR). Two primers, namely 5′CMV (AAGCGGCCTCTGATAACCAAG) and 3′CMV (GAGCAGACTCTCAGAGGATCGG), which anneal to a sequence within the major immediate early gene region of HCMV, were obtained to amplify and quantify the viral genome. In addition, a FAM/TAMRA-labeled probe was used for detection. The viral load of a sample treated with DMSO only served as a negative control. HCMV genome equivalents from peptide-treated viral supernatants were calculated as % of the negative control.

### 3.7. Cytotoxicity Assay

HFF cells were cultivated in 96-well plate with a density of 1.35 × 10^4^ cells per well and incubated at 37 °C for 24 h. The cultured HFF cells were treated with peptides, at concentrations ranging from 0.008 µM to 100 µM, and incubated at 37 °C. On day 5, peptides were refreshed in the culture media and incubation was continued until day 7. A sample without compound (DMSO) served as a negative control, and a sample with 1 µM staurosporine (STP) as a positive control. On day seven, neutral red solution (40 µg/mL, Sigma Aldrich, N2889) was added to cultivated cells and incubated at 37 °C for 24 h, followed by addition of destaining solution (ethanol/water/acetic acid, 50:49:1). Fluorescence was read at excitation/emission at 560/630 nm to quantify the uptake of neutral red.

## 4. Conclusions

Based on a recently reported 29-mer peptide presenting an N-terminal alpha-helical hook-like segment of HCMV pUL53, through which it binds to HCMV pUL50, forming the core nuclear egress complex (core NEC) of HCMV, we fused the pUL53 hook peptide to a cell penetrating peptide (CPP) and a nuclear localization sequence (NLS). The previously established binding affinity to pUL50 of the hook peptide, as well as its inhibitory effect on the pUL50–pUL53 interaction in vitro, was well preserved in the NLS-CPP-Hook fusion peptide. The CPP and NLS moieties were shown to facilitate cellular and nuclear uptake, enabling translocation of the peptide to the inner nuclear membrane. Furthermore, the NLS-CPP-Hook fusion peptide is able to interfere with HCMV infection of cells, as well as with core NEC formation in HCMV-infected cells, while its cytotoxicity at concentrations required for the antiviral effect is negligible. These results further substantiate the notion of the herpesviral core NEC as a potential antiviral target. Thus, while not being an ideal drug candidate by itself, the hook peptide may serve as versatile molecular tool for the molecular characterization of the core NEC, composed of viral proteins pUL50 and pUL53, as well as blueprint for potential inhibitors of the pUL50–pUL53 interaction as candidates for novel anti-herpesviral drugs. Ongoing studies are aimed at optimizing the antiviral activity and stability of the pUL53 hook peptide, as well as at exploring hook peptides derived from homologous core NEC interactions of other herpesviruses, including Epstein–Barr virus (EBV) and Varicella zoster virus (VZV). 

## Figures and Tables

**Table 1:** Peptide sequences, inhibition of the pUL50-pUL53 interaction (IC_50_) and binding to pUL50 (Kd).

Peptide	Sequence	IC_50_ [nM]	Kd [nM]
(Fluo ^a^-)Hook	Ac ^b^(Fluo-Aoa ^c^)-_59_^d^LTLHDLHDIFREHPELELKYLNMMKMAIT_87_-NH_2_	125 ± 16.9 ^e^	228.4 ± 32.22
(Fluo-)NLS-CPP	Ac(Fluo-Aoa)-_18_RSLLRKRRQR_27_-Aoa-_47_YGRKKRRQRRRPP_59_-NH_2_	>10,000	>10,000
(Fluo-)NLS-CPP-Hook	Ac(Fluo-Aoa)-RSLLRKRRQR-Aoa-YGRKKRRQRRRPP-Aoa-LTLHDLHDIFREHPELELKYLNMMKMAIT-NH_2_	46.9 ± 4.7	122.4 ± 20.6

^a^ Fluo, fluorescein; ^b^ Acetyl (Ac), is replaced by (Fluo-Aoa) in fluoresceinylated peptides; ^c^ Aoa, 8-amino-3,6-dioxaoctanoic acid; ^d^ position numbers based on the respective parent proteins; ^e^ standard error based on at least three independent experiments.

**Table 2:** Antiviral (EC_50_) and cytotoxic (CC_50_) effect of peptides.

Peptide	EC_50_ [µM]	CC_50_ [µM]	SI ^b^
Hook peptide	>10	>100	-
NLS-CPP	>10	>100	-
NLS-CPP-Hook	5.6 ± 0.78 ^a^	44.2 ± 6.9	7.9

^a^ Standard deviation based on at least three independent experiments; ^b^ selectivity index (CC_50_/EC_50_).

## Data Availability

Data is contained within the article and Supplementary Materials.

## Supplementary Materials

The following supporting information can be downloaded at: https://www.mdpi.com/article/10.3390/ph15091040/s1, Figure S1: Anti-HCMV activity of the NLS-CPP-Hook peptide measured by virus genome-specific qPCR assay. Figure S2: Analytical data (LC/MS) of synthesized peptides.
