# Supplementary material for: A Peptide Inhibitor of the Human Cytomegalovirus Core Nuclear Egress Complex
Source: Pharmaceuticals (Basel). 2022 Aug 23;15(9):1040. doi: 10.3390/ph15091040 (PMC9505826; doi:10.3390/ph15091040)
Supplement: Supplementary file 1 [file pharmaceuticals-15-01040-s001.zip › pharmaceuticals-1825611-supplementary.pdf]

pharmaceuticals

Supplementary Materials

A peptide inhibitor of the human cytomegalovirus core nuclear egress complex

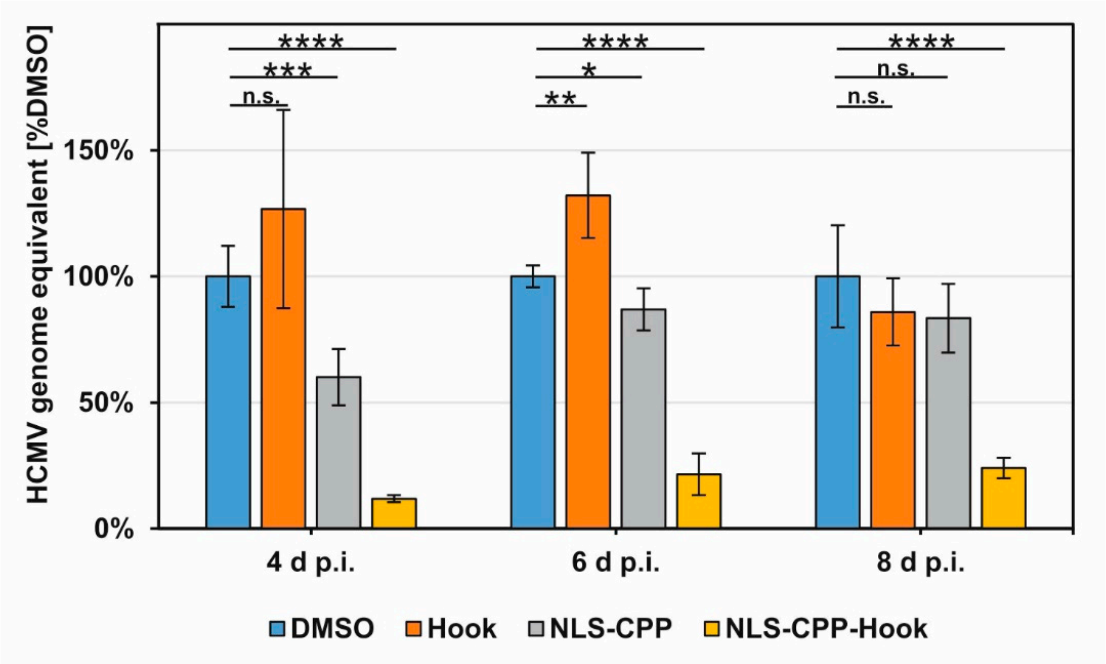

Figure S1. Anti-HCMV activity of NLS-CPP-Hook, measured by virus genome-specific qPCR assay. HFFs were seeded in 12-well plates, used for HCMV infection at a MOI of 0.1 and treated with 6 µM peptide solution. The peptide contained in the culture media was refreshed at 2, 5 and 7 d p.i., and viral supernatants were collected for an assessment of the viral genomic load by quantitative polymerase chain reaction (qPCR) at 4, 6 and 8 d p.i., as a measure of HCMV production and release. HCMV genome equivalents referring to the peptide treatments were calculated as percentage of the DMSO sample. Student's t-test was applied to determine statistical significance (****, $p \leq 0.0001$; ***, $p \leq 0.001$; **, $p \leq 0.01$; *, $p \leq 0.05$; n.s., $p > 0.05$).

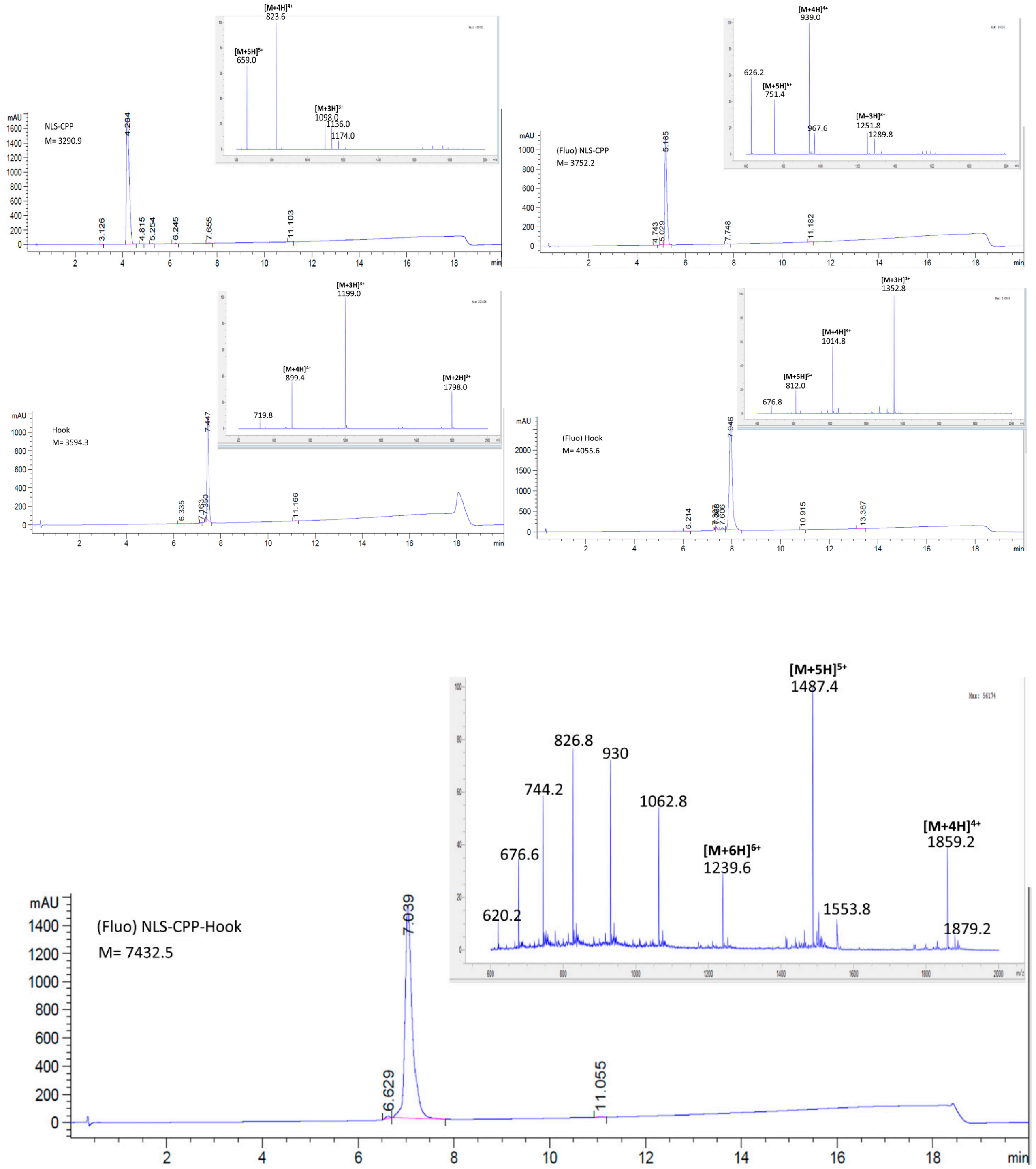

Figure S2. Analytical data (LC/MS) of synthesized peptides.
